# Dual-tDCS Enhances Online Motor Skill Learning and Long-Term Retention in Chronic Stroke Patients

**DOI:** 10.3389/fnhum.2012.00343

**Published:** 2013-01-09

**Authors:** S. Lefebvre, P. Laloux, A. Peeters, P. Desfontaines, J. Jamart, Y. Vandermeeren

**Affiliations:** ^1^Neurology Department, CHU Mont-Godinne UCL, Université catholique de Louvain (UCL)Yvoir, Belgium; ^2^Institute of Neuroscience (IoNS) UCLBrussels, Belgium; ^3^Neurology Department, Clinique universitaire St Luc UCLBruxelles, Belgium; ^4^Neurology Department, C.H.C., Site Saint-JosephLiège, Belgium; ^5^Scientific Support Unit, CHU Mont-Godinne UCLYvoir, Belgium

**Keywords:** transcranial direct current stimulation, motor skill learning, stroke, interhemispheric rivalry, neurorehabilitation

## Abstract

**Background:** Since motor learning is a key component for stroke recovery, enhancing motor skill learning is a crucial challenge for neurorehabilitation. Transcranial direct current stimulation (tDCS) is a promising approach for improving motor learning. The aim of this trial was to test the hypothesis that dual-tDCS applied bilaterally over the primary motor cortices (M1) improves online motor skill learning with the paretic hand and its long-term retention. **Methods:** Eighteen chronic stroke patients participated in a randomized, cross-over, placebo-controlled, double bind trial. During separate sessions, dual-tDCS or sham dual-tDCS was applied over 30 min while stroke patients learned a complex visuomotor skill with the paretic hand: using a computer mouse to move a pointer along a complex circuit as quickly and accurately as possible. A learning index involving the evolution of the speed/accuracy trade-off was calculated. Performance of the motor skill was measured at baseline, after intervention and 1 week later. **Results:** After sham dual-tDCS, eight patients showed performance worsening. In contrast, dual-tDCS enhanced the amount and speed of online motor skill learning compared to sham (*p* < 0.001) in all patients; this superiority was maintained throughout the hour following. The speed/accuracy trade-off was shifted more consistently after dual-tDCS (*n* = 10) than after sham (*n* = 3). More importantly, 1 week later, online enhancement under dual-tDCS had translated into superior long-term retention (+44%) compared to sham (+4%). The improvement generalized to a new untrained circuit and to digital dexterity. **Conclusion:** A single-session of dual-tDCS, applied while stroke patients trained with the paretic hand significantly enhanced online motor skill learning both quantitatively and qualitatively, leading to successful long-term retention and generalization. The combination of motor skill learning and dual-tDCS is promising for improving post-stroke neurorehabilitation.

## Introduction

In the field of stroke neurorehabilitation, motor learning has recently become the focus of a great deal of attention. Motor skill learning is particularly attractive since practice-induced improvement of sensorimotor performance supports development of new aptitudes (skills), which provide the flexibility to adapt to changing conditions. Motor skill learning is defined as a training-induced improvement in motor performance characterized by a shift in the speed/accuracy trade-off that persists over time (Reis et al., 2009; Dayan and Cohen, 2011; Krakauer and Mazzoni, 2011). In other words, motor skill learning requires long-term improvement of both speed and accuracy or improvement of one of these parameters without a simultaneous worsening of the other. Operationally, motor skill learning is demonstrated by improvement over baseline performance during a delayed retention test. Motor skill learning relies on neuroplasticity i.e., this aptitude of the brain to be durably modified by experience and to adapt to changing circumstances (Pascual-Leone et al., 2005). As showed by functional brain imaging (e.g., functional magnetic resonance imaging, fMRI), learning any complex task engages a coordinated “motor learning network” involving multiple brain areas (Krakauer, 2006; Kantak et al., 2010, 2012; Dayan and Cohen, 2011; Krakauer and Mazzoni, 2011; Shmuelof and Krakauer, 2011; Penhune and Steele, 2012). The cerebellum seems necessary for adaptation learning and the primary motor cortex (M1) for learning motor skills (Shmuelof and Krakauer, 2011). For learning sequential motor actions, the striatal system is involved in chunking (concatenating successive movements into “chunks”), the cerebellum acquires internal models optimizing performances and contributing to error correction, and M1 stores the learned sequence (Shmuelof and Krakauer, 2011; Penhune and Steele, 2012). Long-lasting changes in synaptic excitably such as long-term potentiation (LTP) and long-term depression (LTD), protein synthesis, and synaptogenesis in the motor cortex are the neural substrates allowing motor learning (Luft et al., 2004; Monfils et al., 2005). Similarly, LTP-like plasticity of M1 seems to be involved in the formation of motor memory in healthy volunteers (Stefan et al., 2006). Ultimately, the genetic background could determine the potential to achieve successful motor skill learning. E.g., the brain-derived neurotrophic factor (BDNF) gene is one of the multiple genes that influence synaptic plasticity and repair (Bath and Lee, 2006). The BDNF gene shows a common single nucleotide polymorphism leading to an amino acid substitution at position 66 (BDNF Val66Met) that is associated with altered motor plasticity and fMRI patterns (McHughen et al., 2010), less efficient motor learning and reduced responsiveness to non-invasive brain stimulations (Kleim et al., 2006; Reis et al., 2009; McHughen et al., 2010).

Several lines of evidence support the concept that motor learning is an essential component of motor recovery after stroke. First, recovery of motor function after stroke, whether spontaneous or driven by neurorehabilitation, shares common substrates with motor skill learning. Motor skill learning and functional plasticity leading to post-stroke motor recovery share striking similarities in terms of brain networks, fMRI activations, changes in cortical excitability revealed by transcranial magnetic stimulation (TMS) or underlying molecular and genetic substrates (Pascual-Leone et al., 2005; Kreisel et al., 2007; Dayan and Cohen, 2011; Krakauer et al., 2012).

Second, the capacity to achieve at least some forms of motor learning is preserved in most, if not all, stroke patients. For example, use-dependent plasticity, a basic form of motor memory relying on the repetition of a single movement, is conserved in stroke patients (Butefisch et al., 1995; Nelles et al., 2001). Adaptation learning, i.e., the rapid recovery of baseline performance levels under altered experimental conditions such as distorted visual feedback or force field perturbation of ballistic movement, is generally conserved in patients with hemispheric stroke (Takahashi and Reinkensmeyer, 2003). In contrast, patients with damage to the cerebellum or posterior parietal cortex may present specific impairments in adaptation learning (Werner et al., 2010; Palluel-Germain et al., 2011). Motor skill learning appears to be conserved after stroke, as shown by studies using various tasks such as the serial reaction time task, finger sequence tapping, or visuomotor tracking (Carey et al., 2007; Boyd et al., 2010; Bosnell et al., 2011; Dovern et al., 2011; Meehan et al., 2011b). It is worth noting that impairments of specific aspects of motor skill learning may follow injury to the thalamus (Exner et al., 2001), cerebellum (Boyd and Winstein, 2004; Dirnberger et al., 2010; Hatakenaka et al., 2012), or prefrontal cortex (Gomez Beldarrain et al., 1999). Generally, motor skill learning appears to be preserved after stroke, though some aspects may be impaired after damage to specific brain areas.

Third, after a stroke, spontaneous recovery is mediated by a coordinated reorganization of the undamaged cortical areas, their connections and corticospinal projections, subcortical structures (cerebellum, basal ganglia), and spinal cord circuitry (Byrnes et al., 1999; Johansen-Berg, 2007; Xerri, 2012). Recovering motor function after stroke might be conceptualized as learning to use the remaining neural resources to improve motor planning, execution, feedback, and control. Thus, motor recovery after stroke could be a form of motor skill learning. It is still unclear whether post-stroke motor recovery requires the re-learning of damaged/lost motor engrams or the acquisition of new motor skills and internal models. Nevertheless, motor skill learning is undoubtedly one of the key mechanisms underlying the recovery of motor function after stroke.

This is why improving motor skill learning is a major target for neurorehabilitation. It is therefore not surprising that several neurorehabilitation methods have recently been developed on the premise of enhancing motor skill learning (Boyd and Winstein, 2001; Bhatt et al., 2007; Rosser et al., 2008; Abe et al., 2011). Given their capacity to modulate cortical excitability and enhance behavioral performances, non-invasive brain stimulations such as repetitive transcranial magnetic stimulation (rTMS) or transcranial direct current stimulation (tDCS) are particularly attractive as add-on interventions for enhancing post-stroke recovery (Reis et al., 2008). After stroke, deregulated interhemispheric interactions such as unbalanced interhemispheric inhibition from the contralesional M1 toward the ipsilesional M1 influence residual paretic hand function (Murase et al., 2004). Accordingly, rTMS and tDCS can improve residual motor function of the paretic upper limb, likely by rebalancing abnormal interhemispheric interactions (Nowak et al., 2009), enhancing ipsilesional M1 excitability (Hummel et al., 2005; Kim et al., 2006), reducing contralesional excitability (Takeuchi et al., 2008; Zimerman et al., 2012), or doing both (Lindenberg et al., 2010; Bolognini et al., 2011).

Moreover, rTMS can enhance motor learning in stroke patients. High-frequency rTMS applied over the ipsilesional M1 of chronic stroke patients while they trained on a finger sequence tapping task with the paretic hand induced online improvement compared to sham rTMS (Kim et al., 2006). Continuous theta burst stimulation (cTBS, a specific form of rTMS) applied over the contralesional M1 or primary somatosensory cortex (S1) before training on a serial targeting task with the paretic hand improved performance and retention on the following day, as well as improvement on a novel task (Meehan et al., 2011a). However, broad use of rTMS in clinical settings is hindered by several factors: (1) risk of inducing a seizure especially in patients with a brain lesion (Nowak et al., 2006; Lomarev et al., 2007), (2) relative difficulty of use, (3) uncomfortable sensations, (4) lack of convincing sham rTMS, and (5) price of rTMS devices. Given its safety (Nitsche and Paulus, 2001; Merrill et al., 2005), portability, user-friendly and patient-friendly features, existence of convincing sham stimulations (Gandiga et al., 2006), and lower price, tDCS seems more likely than rTMS to rapidly become a therapeutic adjuvant in neurorehabilitation. In healthy volunteers, anodal tDCS over the contralateral M1 improves the speed/accuracy trade-off on a visuomotor task involving serial pinch contractions, enhances motor skill learning and long-term retention (Reis et al., 2009). In chronic stroke patients, a recent study showed that cathodal tDCS over the contralesional M1 improved motor skill learning on a finger sequence tapping task, as well as overnight retention (Zimerman et al., 2012).

To date, evidence supporting motor skill learning improvements has been mostly based on online improvement or very specific tasks restricted to stroke patients with an excellent motor recovery (e.g., able to perform complex finger sequence task). It is only recently that studies have used a modern definition of motor skill learning (i.e., shift of the speed/accuracy trade-off) and/or investigated long-term retention (Reis et al., 2009; Meehan et al., 2011a). Therefore, before implementing non-invasive brain stimulations as therapeutic adjuvants in stroke neurorehabilitation, it is mandatory to test the impact of non-invasive brain stimulation on long-term retention of motor skills in stroke patients and to develop motor skill learning tasks (i) involving a speed/accuracy trade-off, (ii) requiring the activation of the whole upper limb in complex sequences of movements, and/or (iii) having clearer ecological relevance to daily life activities.

The present study tested the hypothesis that dual-tDCS applied in chronic stroke patients while they learned a new motor skill with the paretic upper limb enhances long-term retention of the motor skill (primary aim). Secondary aims were to test whether dual-tDCS (i) improves online motor skill learning, (ii) modifies the quality of motor skill learning by shifting the speed/accuracy trade-off, and (iii) allows generalization of improvement beyond the learned motor skill.

## Patients and Methods

### Population

**Figure 1:**
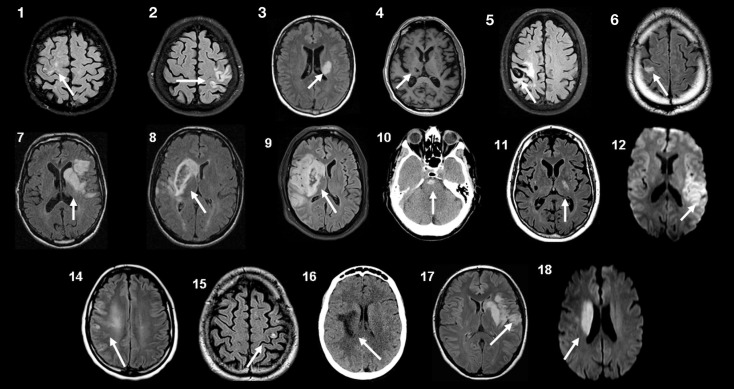
**Brain imaging**. Magnetic resonance imaging (MRI) or computed tomography (CT) scans at the level of the stroke for each patient. Patients 10 and 16 had CT scans, patient 4 had a T_1_-weighted MRI, patients 12 and 18 had Diffusion-Weighted Imaging (DWI), and all others had FLAIR T_2_-weighted MRI. Patients 4 and 5 had an intracerebral hemorrhage. Patients 8 and 9 had a slight secondary hemorrhagic transformation. Patients 1, 15, and 17 had at least one other lesion compatible with a previous, minor stroke. Patient 6 had associated leukoaraiosis and small chronic subcortical infarcts. Patient 4 had small chronic subcortical lacunar infarcts. For patient 13, the MRI scans were not retained in the patient’s medical folder, but a detailed neuroradiological report permitted localization of the lesion (Table 1).

The protocol was approved by the local Ethical Committee (Comité d’éthique médicale, CHU Mont-Godinne, UCL) and was conducted according to the recommendations of the Helsinki declaration. Eighteen patients with a chronic stroke provided written informed consent after reviewing the following inclusion criteria: (1) being a chronic (>6 months) stroke patient aged 18–80 years, (2) presenting a chronic motor deficit in the upper limb, (3) having an hemispheric vascular brain lesion demonstrated by cerebral imaging (Figure 1). Exclusion criteria were: being unable to perform the task or to understand instructions, presence of intracranial metal, epilepsy, alcoholism, pregnancy, cognitive impairment, or psychiatric disorder. Ten patients had an ischemic cortical stroke, six a subcortical ischemic stroke, and two (#4 and 5) had an intracerebral hemorrhage (Figure 1; Table 1). Some patients had more than one type of stroke. Residual dexterity was quantified with the baseline Purdue Pegboard Test (PPT, see below), residual manual ability with the ABILHAND scale (Penta et al., 2001; Table 1), and the overall degree of disability with the modified Rankin Scale (mRS; Bonita, 1988).

**Table 1:** **Baseline patient characteristics**.

Stroke Patients	Gender	Age	Time since stroke (years)	Main stroke lesion	Additional vascular lesions	Dominant hand	Paretic Hand	Paretic handPPT (*n*)	Non-paretic handPPT (*n*)	Paretic hand MaxHF (kg)	Non-paretic handMaxHF (kg)	ABILHAND (logits)	mRS
1	F	65	0.5	C	SC-C	R	L	1.3	10.7	16	28	1.5	3
2	M	67	2	C	–	R	R	10.3	12.7	36	45	2.3	2
3	M	64	4	SC	–	R	R	6.3	14.3	38	42	1.5	2
4	M	61	6	SC (H+)	1 SLI	L	L	1	13.7	34	41	0.3	3
5	M	61	4	C (H+)	–	R	L	6.3	11.3	36	51	0.3	2
6	M	56	3	C	LK, SSIs	R	L	7.7	12.3	30	41	3.9	2
7	M	69	4	C	–	R	R	7.3	10.7	53	38	1.5	2
8	M	49	3	C	–	R	L	7	16.3	28	47	1.7	2
9	M	63	1	C	–	R	L	3	13.7	26	35	1.9	2
10	M	65	0.8	SC	–	R	L	13	14.7	49	55	5.5	1
11	M	70	3	SC		R	R	9	11.3	35	47	4.3	1
12	M	55	1	C	–	R	R	9	11.7	55	50	1.9	2
13	F	68	3	SC	–	R	R	10.3	13	22	22	2.4	2
14	F	36	2	C	–	R	L	9.7	13.7	17	19	1.3	2
15	M	79	3	C	SC-C	R	R	2.3	8	28	40	1	3
16	F	61	3	SC	–	R	L	0	15.3	0	29	1.2	3
17	F	35	3	C	SC-C	R	R	17.3	17.3	33	31	4.4	2
18	F	61	1	SC	–	R	L	7	17.3	21	25	1.6	3
Mean ± SD		61 ± 9	2.6 ± 1.5					7.1 ± 4.5	13.2 ± 2.5	31 ± 14	38 ± 11	2.1 ± 1.5	2 ± 1

*M, male; F, female; SC, subcortical stroke; C, cortical stroke; H+, intracerebral hemorrhage; R, right; L, left; PPT, Baseline Purdue Pegboard Test score; *n*, number of pegs inserted in 30 s (mean of three trials); MaxHF, Maximal hand grip force; mRS, modified Rankin Scale; kg, kilograms. In addition to their main stroke lesion, five stroke patients presented additional vascular lesions: SSIs, small subcortical infarctions; SLI, subcortical lacunar infarctions; LK, leukoaraiosis*.

### Study design

**Figure 2:**
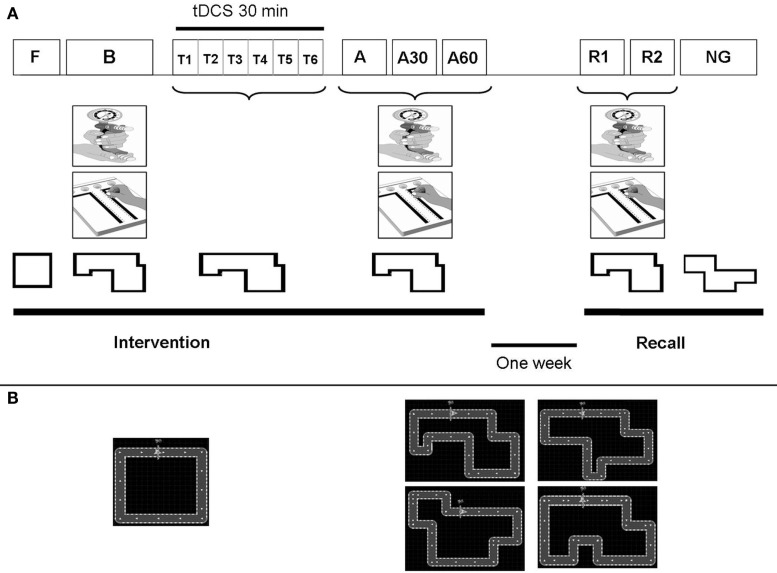
**Study design**. **(A)** Study design: Patients participated in two intervention sessions, each of which was followed by a Delayed Recall session. Intervention sessions comprised 6 periods: Familiarization (F), Baseline (B), Training (T) and Immediate (A1), 30 min (A2), and 60 min (A3) tests. Delayed Recall sessions comprised 3 periods: Recall 1 (R1), Recall 2 (R2), and New Circuit Game (NG) tests. During F, patients performed an easy circuit over 1 min. During B, A1, A2, A3, R1, and R2, patients performed the Purdue pegboard test (PPT), Maximal hand grip force (MaxHF), and the “circuit game” with the specific circuit assigned to that session. During T, patients performed five blocks of six trials of the “circuit game” (with the specific circuit assigned to that session). During these Training, patients received 30 min of dual-tDCS or sham, based on their randomization order. During NG, patients performed a New Circuit Game of the same length and difficulty. **(B)** Left, square circuit used for Familiarization; Right, the four circuits of identical length and complexity used for motor skill learning, and New Circuit Game.

**Figure A1:**
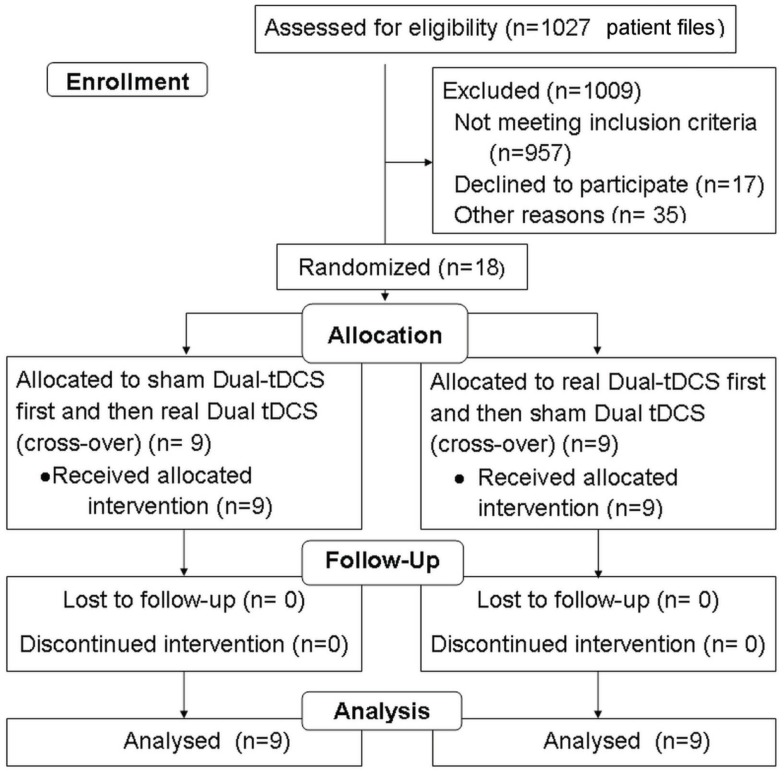
**CONSORT flow diagram**. Method of randomization: An experimenter established an inclusion list, attributing the Eldith^®^ codes for dual-tDCS and sham to the first and second session in pseudo-randomized, balanced order for each successive patient. A second experimenter applied these codes blindly, and patients were not aware of their treatment, such that dual-tDCS was delivered in a double-blind fashion. Since it was the first time that this paradigm was used in stroke patients to induce long-term retention after motor skill learning, no power analysis was performed.

The study was a randomized, placebo-controlled (sham), double-blind, cross-over trial involving two blocks of two sessions each (Figure 2A; Figure A1 in Appendix). Each block consisted of motor skill learning under dual-tDCS or sham dual-tDCS in the first session (Intervention) and a retention test 1 week later in a second session (Delayed Recall). The interval between Delayed Recall of session 1 and Intervention of session 2 was at least 1 week (1.4 ± 0.7 week).

The Intervention session comprised the six successive periods. (1) The Familiarization involved performing 1 min of habituation on a simple version of the motor skill learning task (a square circuit) with the paretic hand. (2) The Baseline included (i) measuring the maximal grip force of each hand with a Jamar dynamometer over three trials to determine mean maximum hand grip force (MaxHF), (ii) performing the PPT three times with each hand to determine the mean number of pegs placed (Gallus, 2003), and (iii) performing the motor skill learning task (see below) with the paretic hand during two blocks of 30 s, with 30 s of rest between blocks. (3) The Training involved learning the task by performing the motor skill with the paretic hand over 30 min, alternating 30-s blocks of training and rest, while receiving dual-tDCS (Stagg et al., 2011). (4–6) The Early Recall tests were conducted immediately, 30 and 60 min after completing training and involved measurement of (i) MaxHF, (ii) PPT, and (iii) performance of the motor skill with the paretic hand over 5 min, alternating 30-s blocks of testing and rest.

The Delayed Recall session was performed 1 week later and comprised three periods: (1) Recall 1 and (2) Recall 2 which were identical to the Early Recall tests; and (3) New Circuit Game which involved performing an alternative version of the motor skill over 5 min, to test for a generalization effect on a novel, untrained circuit.

### Motor skill learning

Stroke patients trained on the “circuit game,” which induces motor skill learning and retention in healthy volunteers (Lefebvre et al., 2012). Patients were comfortably seated and held a computer mouse on a desk with their paretic hand. A complex circuit was displayed on a computer screen (Figure 2B). The instructions were: “Use the computer mouse to move the pointer as fast and accurately as possible over the circuit. Accurately means keeping the cursor within the track. Improvement during training is expected.” To motivate the patients, a high score reflecting their error and velocity during the previous block was displayed on the screen during rest periods. Behavioral data (error and velocity) were stored and analyzed off-line. Four different circuits of identical length and complexity (i.e., equal number of corners and segments arranged in a different order) were used during the two Intervention sessions and the two New Circuit Game tests (Figure 2B). A pilot experiment in another group of stroke patients (*n* = 7, age: 62 ± 5.5 years, four had a cortical lesion and three a subcortical lesion, all presented chronic upper limb paresis) demonstrated that these circuits were of equal difficulty. The seven chronic stroke patients of the pilot group performed each circuit during 5 min in a random order, after breaks of 5 min. The mean velocity, error, and laps number were not statistically different between the four circuits (*p* = 0.07; *p* = 0.37; *p* = 0.28 respectively).

To quantify performance on the motor skill, error, and velocity were extracted and combined in a performance index (PI). Error was defined as the surface area between the pointer’s trajectory and the midline of the track. Velocity and error were averaged in bins of 3 s, resulting in 10 values for each 30-s Training block. Normalized mean error (Pe = *a*/subject mean error) and normalized mean velocity (Pv = subject mean velocity/*b*) were used to compute the PI, which is designed to increase when error diminishes and/or when velocity increases (PI = Pv * Pe). “*a*” and “*b*” are constant values of error and velocity derived from the pilot group of seven other stroke patients (see Appendix 1). Increase in PI reflects enhanced motor skill performance, defined as an improvement of the speed/accuracy trade-off.

To quantify evolution of motor skill learning over time, a learning index (LI) was calculated for each block. The evolution of the PI from Baseline was expressed as LI = [(PI − PI baseline)/PI baseline] × 100. An increment of LI over time reflects an improvement in performing the motor skill relative to Baseline (i.e., motor skill learning). The LIs from five consecutive Training blocks were grouped and used for statistical analysis. As reported previously (Lefebvre et al., 2012), three main patterns of evolution can be observed: (1) no learning (i.e., lack of change or worsening), (2) motor skill learning with a fit pattern (i.e., improvement of the LI limited by an opposite evolution of Pv and Pe), and (3) motor skill learning with a shift pattern (i.e., improvement of the LI due to improvement of both Pe and Pv or to an improvement of one parameter without a concomitant deterioration of the other). Since the shift pattern demonstrates a clear improvement of the speed-accuracy trade-off, it reflects more efficient motor skill learning than the fit pattern (Lefebvre et al., 2012).

For the New Circuit Game, PIs from five consecutive blocks were grouped and used to compare generalization of motor performance on an untrained circuit (real-dual-tDCS versus sham). Since the New Circuit Game consisted in only five blocks, no LI (reflecting changes) could be computed but only online performance (PI).

### Dual-tDCS

An Eldith DC-Stimulator^®^ (NeuroConn, Ilmenau, Germany) delivered dual-tDCS via two soaked (NaCl 0.9%) electrodes (35 cm^2^). The hot spot eliciting consistent movements in the contralateral hand was localized using a Magstim 200^2^ (Magstim Company, UK) with a figure-of-eight coil to localize left and right primary motor cortices (M1). For patients 15 and 16, the Magstim 200^2^ was not available, and M1 were localized using the international 10/20 EEG system where C3 and C4 correspond to M1. The anode electrode was positioned over the ipsilesional M1 and the cathode electrode over the contralesional M1. For dual-tDCS, stimulation at 1 mA (fade-in/out 8 s) was applied over 30 min. For sham dual-tDCS, a short current up-ramp (8 s fade-in) was followed by 30 s of direct current to induce similar scalp sensations, then by 8 s of current fade-out. The Eldith^®^ codes corresponding to tDCS and sham tDCS were selected by an experimenter to establish an inclusion list with a pseudo-randomized, balanced order (see the CONSORT flow diagram in Figure A1 in Appendix). These codes were used in a double-blind fashion by a second experimenter. None of the patients reported adverse effects with tDCS.

### Statistical analysis

The primary outcome measure was the amount of motor skill retention at 1 week (LI of Recall 1), compared between real and sham dual-tDCS with a paired *t*-test. For the evolution of the “circuit game” during Training and up to 60 min after, repeated measures analyses of variance (rmANOVA) were used to explore the effect of Stimulation (dual-tDCS, sham) and Time (Baseline, Training, Immediate, 30, 60 min). For *post hoc* analyses, paired-sampled *t*-tests were used to compare each LI value between Stimulation (dual-tDCS, sham). Paired-sample *t*-test were also used to compare mean LI and PI of Recall 2 and the New Circuit Game.

For PPT and MaxHF, rmANOVA were performed for the Intervention session; paired-sampled *t*-tests were used for *post hoc* analyses. Paired-sample *t*-tests were also performed between Baseline, Recall 1, and Recall 2.

Correlations analyses were performed to determine whether baseline clinical characteristics (age, mRS, and ABILHAND score) predicted the individual percentage of LI improvement at Recall 1 (the primary outcome measure) after dual-tDCS. In order to disclose whether the stroke localization (cortical/subcortical) influenced the responsiveness to real-dual-tDCS, a Student’s *t*-test was calculated to seek for a difference in the LI of Recall 1 between the subgroups (cortical/subcortical).

All statistical tests (both for Recall comparisons and *post hoc* analyses of the rmANOVAs) were two-tailed, and corrected for multiple comparisons (Bonferroni) i.e., each observed *p*-value was multiplied by the number of comparisons performed. A *p*-value of 0.05 was considered statistically significant. Statistical analyses were performed using SPSS^®^ 15.0 (SPSS Inc., Chicago, IL, USA).

## Results

### Primary outcome: Impact of dual-tDCS on long-term motor skill retention

**Figure 3:**
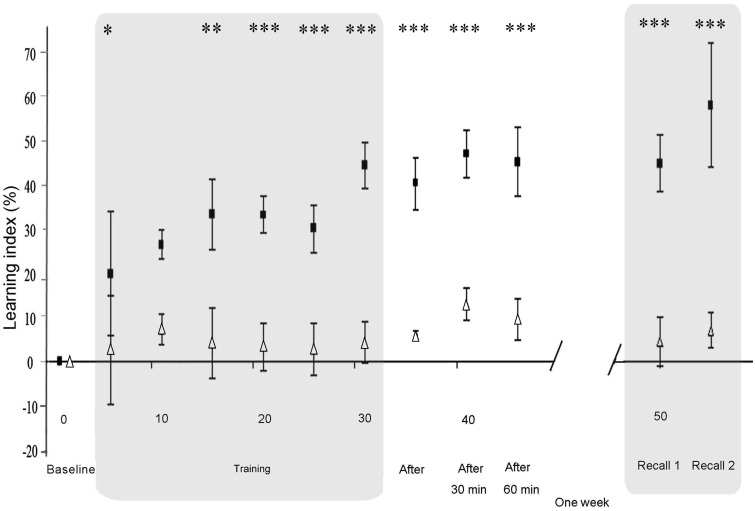
**Differential evolution of motor skill learning under sham and dual-tDCS**. Evolution of the Learning Index (LI), expressed as a percentage change from Baseline during the Intervention session [Baseline, Training, Immediate (After), 30, and 60 min] and Delayed Recall session (Recall 1 and Recall 2). LI is plotted as the mean ± SD of five consecutive blocks of the “circuit game.” LI was significantly improved under dual-tDCS compared to sham from the third block of Training until the end of testing. Numbers on the *X*-axis refer to blocks of the “circuit game.” White triangles, sham; black squares, dual-tDCS. *p < 0.05, **p < 0.005, ***p < 0.001 [all *p* values corrected for multiples comparisons (Bonferroni)].

One week after Training (Recall 1), the motor skill LI after dual-tDCS (44% ± 25, mean ± SD) was statistically superior to that observed after sham (4% ± 24; *p* < 0.001; Figure 3). A similar effect was observed at Recall 2 (*p* < 0.001). Moreover, there was a clear performance improvement between Recall 1 and Recall 2 (+13%) 1 week after dual-tDCS and only a modest improvement after sham (+3%). However, this difference did not reach statistical significance (*p* = 0.11). The order of interventions (real-dual-tDCS first or second) did not influence these results (*p* = 0.10: no order effect).

One week after sham dual-tDCS, at Recall 1, 7 out of 18 stroke patients exhibited LI degradation (Table A1 in Appendix). The other 11 patients demonstrated a significant retention of the motor skill (i.e., a positive LI value): 6 presented a fit pattern and 5 a shift pattern. In sharp contrast, 1 week after real-dual-tDCS, the 18 stroke patients showed a consistent retention of the LI improvement: 7 patients presented a fit pattern and 11 a shift pattern (Table A1 in Appendix).

### Impact of dual-tDCS on online motor skill learning and early recalls

Baseline PI values were not statistically different between the nine stroke patients starting with real-dual-tDCS and those starting with sham (*p* = 0.23). rmANOVA on the LI during Training and up to 60 min after showed a significant interaction between “Time” and “Stimulation” (*p* < 0.001) suggesting that dual-tDCS led to greater online motor skill learning and Early Recalls than sham. rmANOVA also showed a significant effect of “Stimulation” (*p* < 0.001), suggesting that dual-tDCS-induced a greater online motor skill learning since the third block of Training (*p* = 0,002; Figure 3) and a significant effect of “Time” (*p* < 0.001), suggesting that stroke patients generally improved regardless of intervention. *Post hoc* analyses demonstrated that dual-tDCS led to a significantly greater and more rapid improvement than sham (Figure 3). No order effect was found between the two arms of the cross-over design (*p* = 0.19).

Under sham dual-tDCS, eight out of 18 stroke patients exhibited LI degradation during the Training period. The remaining 10 patients improved, as shown by the evolution of their velocity and error: 7 of them presented a fit pattern and three a shift pattern (Table A1 in Appendix). In sharp contrast, the 18 stroke patients showed consistent LI improvement after dual-tDCS (i.e., they all achieved motor skill learning). Eight patients presented a fit pattern and 10 a shift pattern.

**Figure 4:**
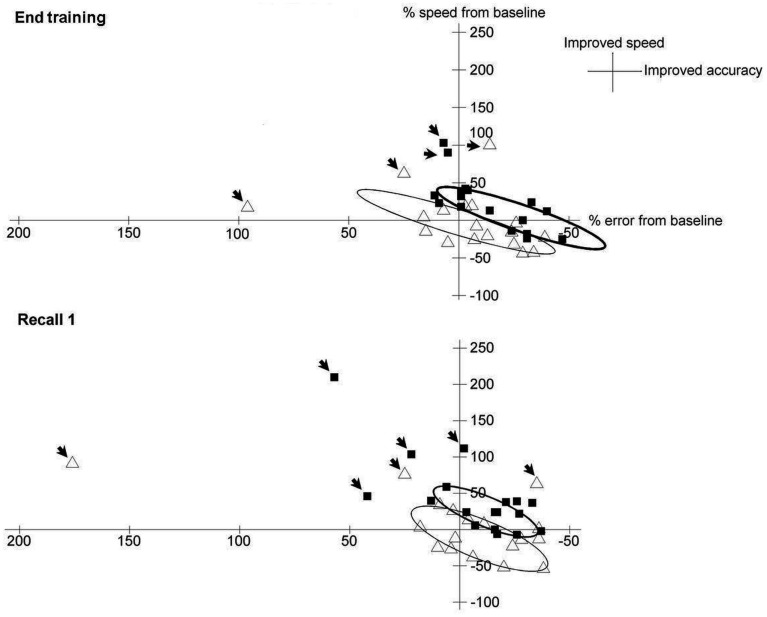
**Trade-off between error and velocity under sham and dual-tDCS**. Matlab^®^ (The MathWorks) was used to generate the scatter plots and ellipses. Scatter plot of the trade-off between error (*X*-axis) and velocity (*Y*-axis), expressed as percentage change from Baseline, for each patient after dual-tDCS (black squares) or sham (white triangles) at the end of Training (upper panel) and at Recall 1 (lower panel). The ellipses [contain 90% of the values, outliers (arrows)] show that both error and velocity improved more after dual-tDCS than sham, demonstrating a shift of the speed/accuracy trade-off, as expected in efficient motor skill learning. Moreover, whereas the ellipse for sham is roughly centered over the equilibrium point, the ellipse for dual-tDCS is clearly shifted from this point, in line with a shift of the speed-accuracy trade-off.

To better depict the trade-off between error and velocity, patients’ respective changes from Baseline under dual-tDCS and sham were extracted from the end of the Training period (last five blocks of Training) and from Recall 1 and were displayed as scatter plots in Figure 4 (see also Table A1 in Appendix). The ellipses (computed to contain 90% of the values; Matlab, the Mathworks^®^ R2009b) graphically emphasize that both error and velocity improved more after dual-tDCS than after sham, consistent with an enhanced shift of the speed/accuracy trade-off.

### Generalization to a new circuit game

After completing Recall 1 and 2, the stroke patients performed a New Circuit Game during 5 min on another circuit of identical length and difficulty (see Figure 2), to test for a generalization effect on a novel, untrained circuit. The PIs from the five consecutive blocks were grouped to compare generalization of motor performance on a new, untrained circuit between real-dual-tDCS and sham. The PI was significantly greater after real-dual-tDCS (1.55 ± 1.01) than after sham (1.38 ± 0.87; *p* = 0.045).

### Generalization to dexterity and force in the paretic hand

Baseline PPT scores were not statistically different between the nine stroke patients starting with real-dual-tDCS and the nine other patients starting with sham (*p* = 0.34). The PPT score of the paretic hand improved over time after dual-tDCS (e.g., +1.4 pegs inserted in 30 s, +19% at 60 min, Table 2), but not after sham (+0 pegs in 30 s, 0%). rmANOVA showed a significant “Time × Stimulation” interaction (*p* = 0.001), suggesting that dual-tDCS had an impact on the evolution of the PPT score across the Intervention session. *Post hoc* analyses demonstrated that at Baseline, there was no significant difference between dual-tDCS and sham (*p* = 0.1) whereas there was a statistically significant difference between dual-tDCS and sham (*p* = 0.009) at the last Early Recall (60 min).

**Table 2:** **Changes in PPT and MaxHF for paretic and non-paretic hands**.

Task	Sham dual-tDCS						Real-dual-tDCS					
	Baseline	After	After 30 min	After 60 min	Recall 1	Recall 2	Baseline	After	After 30 min	After 60 min	Recall 1	Recall 2
PPT (PH)	8,0 ± 3.6	7,8 ± 3.5	8,1 ± 3.9	8,0 ± 4.1	8,2 ± 4.0	8.2 ± 4.3	7,4 ± 4.4	8,2 ± 4.4	8,8 ± 4.1	8,8 ± 4.3	8,4 ± 4.0	8,7 ± 3.9
PPT (n-PH)	13,5 ± 2.7	13,2 ± 3.3	13,6 ± 2.9	13,7 ± 3.0	13,5 ± 3.2	13,4 ± 3.3	13,1 ± 2.7	13,2 ± 2.8	14,0 ± 2.7	13,9 ± 2.8	13,2 ± 3.1	13,2 ± 2.9
MaxHF (PH)	32,6 ± 12.1	31,4 ± 12.1	32,7 ± 13.2	34,2 ± 13.2	34,2 ± 13.2	33,4 ± 13.9	34,0 ± 12.4	33,5 ± 13.2	34,3 ± 13.3	33,8 ± 13.0	34,1 ± 12.0	33,7 ± 11.7
MaxHF (n-PH)	37,6 ± 10.6	37,4 ± 11.3	38,1 ± 12.1	39,1 ± 12.1	39,0 ± 11.9	38,9 ± 11.9	38,5 ± 11.6	38,0 ± 11.6	39,0 ± 12.0	38,7 ± 11.9	39,8 ± 10.3	40,0 ± 11.7

*Group data (mean ± SD) for the Purdue pegboard test (PPT) and Maximal hand grip force (MaxHF) for each evaluation period (Baseline, Immediate, 30 min, 60 min, Recall 1, and Recall 2) for paretic hand (PH) and non-paretic hand (n-PH) receiving dual-tDCS and sham*.

One week after dual-tDCS, PPT scores remained significantly improved at Recall 1 (+0.8 pegs in 30 s, +13%, *p* = 0.021) and Recall 2 (+1.2 pegs in 30 s, +17%, *p* < 0.001) compared to Baseline but not after sham (Recall 1, *p* > 0.9; Recall 2, *p* > 0.9; Table 2). No order effect was found between the two arms (*p* = 0.37).

Baseline MaxHF were not statistically different between the nine stroke patients starting with real-dual-tDCS and those starting with sham (*p* = 0.73). For the paretic MaxHF, rmANOVA demonstrated a significant effect of “Time” (*p* = 0.008), but not of “Stimulation” (*p* = 0.1), nor of the “Time × Stimulation” interaction (*p* = 0.2). Furthermore, *post hoc* analyses demonstrated that there were no statistical difference on MaxHF between sham and dual-tDCS at any time (Baseline, Early Recalls, Recall 1, and Recall 2). This suggests a slight progressive improvement of MaxHF, independent of the type of stimulation (Table 2).

### Dexterity and force in the non-paretic hand

For the PPT score of the non-paretic hand, rmANOVA demonstrated a significant effect of “Time” (*p* = 0.009), but neither of “Stimulation” (*p* = 0.9) nor of the “Time × Stimulation” interaction (*p* = 0.3), suggesting a slight progressive improvement, independent of the type of stimulation. No significant change in PPT score was observed 1 week later (Table 2). The non-paretic MaxHF hand remained unchanged during Intervention and at Delayed Recall (Table 2).

### Correlation analyses

Correlations analyses were performed to determine whether baseline clinical characteristics predicted the individual percentage of LI retention at Recall 1 after real-dual-tDCS. The patient’s age, mRS, and ABILHAND scores did not correlate significantly with LI improvement at Recall 1 (*p* = 0.68, *p* = 0.55, and *p* = 0.95, respectively), nor did the localization of the stroke [cortical versus subcortical: *p* = 0.43 (Student’s *t*-test)].

### Carry-over effect

In order to determine whether a carry-over effect could be observed with the cross-over design, statistical comparisons were performed for each parameters (PI, PPT, and MaxHF) between the Baselines of the first and second Intervention separately for the two patient’s groups. For the nine patients who received sham dual-tDCS during the first Intervention, there was no statistically significant difference between the Baseline performances of Intervention 1 (sham) and 2 (dual-tDCS; PPT: *p* = 0.53, MaxHF: *p* = 0.18, and PI: *p* = 0.06).

For the nine patients who started with real-dual-tDCS as first Intervention, there was no statistically significant difference between the Baseline performances of Intervention 1 (dual-tDCS) and 2 (sham) for the PPT (*p* = 0.07) and MaxHF (*p* = 0.61). However, Baseline PI of Intervention 2 (i.e., 1 week after the Delayed Recall session that followed real-dual-tDCS) was significantly superior when compared to the Baseline PI of Intervention 1 (*p* < 0.001).

## Discussion

The main findings of this experiment were that 30 min of dual-tDCS applied bilaterally over M1 in chronic stroke patients while they learned a complex motor skill with the paretic hand (i) rapidly and significantly enhanced online motor skill learning, (ii) enhanced the *quality* of motor skill learning by increasing the shift of the speed/accuracy trade-off, (iii) successfully translated online improvement into long-term retention of the motor skill, and (iv) induced a generalization of performance improvement to untrained tasks, such as digital dexterity and an alternative version of the motor skill.

### Dual-tDCS enhances the amount and quality of online motor skill learning in stroke patients

Under sham dual-tDCS, 10 of 18 chronic stroke patients (56%) achieved online motor skill learning, while eight (44%) showed online deterioration of performance. Of those who deteriorated, four steadily worsened from the beginning and four started to improve but worsened later. This online deterioration of performance could be due to fatigue, lack of attention, or inability to engage the motor skill learning network, possibly secondary to an imbalance of interhemispheric excitability (see below). Only one patient (#12) presented positive retention of the motor skill with a fit pattern after 1 h and 1 week, suggesting that he achieved off-line motor skill learning despite online worsening.

In sharp contrast, under dual-tDCS, all the stroke patients (100%) achieved online motor skill learning, showing a dramatic improvement by the end of the Intervention session (dual-tDCS: +44%, sham: +4%). Moreover, dual-tDCS considerably improved the efficiency of online motor skill learning, since the LI was already statistically increased under dual-tDCS compared to sham after a few blocks of training (Figure 3). This translated into superior retention of the motor skill 60 min and 1 week after dual-tDCS.

The current experimental paradigm was designed to involve a speed/accuracy trade-off for evaluating motor skill learning. This permitted to demonstrate that, in addition to enhancing the amount and speed of online motor skill learning as well as long-term retention, dual-tDCS improved the *quality* of motor skill learning. Among the 10 stroke patients (56%) who achieved online motor skill learning under sham dual-tDCS, only three (17%) adopted the most efficient pattern of motor skill learning, the shift pattern; the remaining seven patients (39%) followed a fit pattern. In sharp contrast, all of the stroke patients achieved online motor skill learning under dual-tDCS, 10 (56%) with a shift pattern and eight (44%) with a fit pattern. Thus, compared to sham, dual-tDCS also improved motor skill learning *quality* through increased shift of the speed/accuracy trade-off (i.e., more efficient motor skill learning), which translated into a successful long-term retention.

Could the observed online and 1-h post-intervention improvements “simply” result from modified excitability driven by dual-tDCS? In healthy volunteers, 13 min of anodal tDCS can induce changes in corticomotor excitably lasting up to 90 min (Nitsche and Paulus, 2001), and 20 min of cathodal tDCS changes up to 180 min (Di Lazzaro et al., 2012). In stroke patients, 20 min of tDCS can modulate corticomotor excitability up to 60 min after intervention (Zimerman et al., 2012) and enhance motor performance up to 30 min (Hummel et al., 2005). In this type of experiment, it is by definition not possible to disentangle online motor skill learning enhancement and early re-tests from “simple” motor performance improvement driven by tDCS. However, in the current experiment, the facts that (i) dual-tDCS translated into successful long-term retention of the motor skill and (ii) there was no off-line improvement unambiguously demonstrate that dual-tDCS indeed enhances online motor skill learning. A similar reasoning applies for the observed improvements of digital dexterity, although there was a limited exposition to the PPT compared to motor skill learning. Since the PPT remained improved 1 week after dual-tDCS but not after sham, this long-lasting enhancement cannot be attributed to tDCS online effects or after-effects.

### Successful long-term retention in stroke patients following online enhancement of motor skill learning under dual-tDCS

This is the first demonstration that enhancement of online motor performance induced by dual-tDCS in stroke patients translated into successful long-term retention of the motor skill learned with the paretic hand, which is a fundamental step forward for neurorehabilitation. Indeed, if a single-session of dual-tDCS enhances online motor skill learning and leads to long-term retention of a complex motor skill, then repeated sessions of dual-tDCS combined with neurorehabilitation are likely to improve durably motor recovery. Interestingly, whereas the retention test was not designed to asses continued motor skill learning, the improvement observed between Recall 1 and Recall 2 was larger after dual-tDCS (+13%) than after sham (+3%). This suggests that recall of the motor skill acquired under dual-tDCS could reactivate mechanisms that place the brain in an optimal state for subsequent motor skill learning. However, this hypothesis remains to be tested formally. Similarly, whether repeated sessions of motor skill learning coupled with dual-tDCS in stroke patients leads to cumulative improvement, as previously observed in healthy volunteers (Reis et al., 2009), should also be tested.

There was no correlation between the baseline clinical characteristics of the stroke patients (age, mRS, ABILHAND, whether the lesion was cortical or subcortical) and the amount of long-term retention. Since this cohort of 18 patients with mixed stroke subtypes matches well “real-life” stroke patients, the present results are encouraging for a broad implementation of dual-tDCS as add-on therapy for neurorehabilitation in a large range of stroke patients.

### Generalization of performance improvements and carry-over effect

After the two Recall trials, patients practiced a New Circuit Game during 5 min, with one of the alternative circuit versions of identical length and complexity. Performance on this new, untrained circuit was significantly better after real-dual-tDCS than after sham. Thus, dual-tDCS-induced a greater generalization of motor performance improvement than sham, which persisted 1 week after intervention. Alternatively, recalling the motor skill learned under dual-tDCS 1 week before may have placed the motor system in an optimal state for inducing a generalization of performance improvement to an untrained version of the motor skill.

Dual-tDCS had no impact on paretic hand’s grip force. Conversely, digital dexterity of the paretic hand was greatly enhanced immediately after Training under dual-tDCS and kept improving up to +19% 60 min after. This suggests that combination of motor skill learning and dual-tDCS may lead to generalized improvement on complex or demanding tasks such as the PPT. Alternatively, the protracted improvement of the paretic hand’s digital dexterity may reflect a subtle and delayed after-effect of tDCS (Lefebvre et al., in press). Interestingly, although there was a slight drop in digital dexterity 1 week later, the enhancement remained significant at Recall 1 and showed a trend toward improvement from Recall 1 (+13%) to Recall 2 (+17%) after dual-tDCS but not after sham. Thus, dual-tDCS not only enhanced online motor skill learning and long-term retention with the paretic hand but also led to generalization of motor performance improvements with an alternative version of the motor skill, as well as a lasting improvement of digital dexterity.

**Figure A2:**
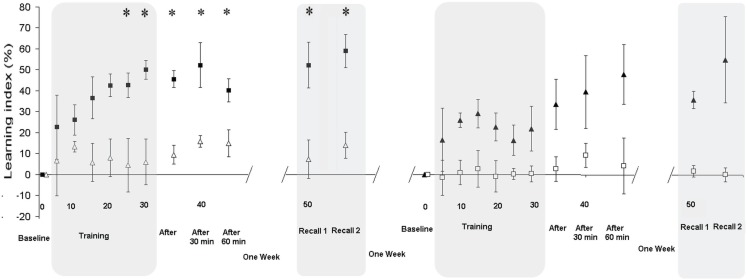
**Split groups analysis**. The left panel displays the first session (Intervention and Recall) separately for the nine stroke patients randomly allocated to real-dual-tDCS as the first intervention (black squares) and for the nine others allocated to sham (white triangle); i.e., as if the study had assumed a parallel-group design. The LI was significantly improved under dual-tDCS compared to sham from the fifth block of Training (see Appendix 2). The right panel displays the second session (Intervention and Recall) for the two groups of stroke patients [sham (white squares) or dual-tDCS (black triangle) as the second intervention]. Evolution of the Learning Index (LI) is expressed as a percentage change from Baseline during the Intervention session [Baseline, Training, Immediate (After), 30, and 60 min] and Delayed Recall session (Recall 1 and Recall 2). LI is plotted as the mean ± SD of five consecutive blocks of the “circuit game.” *p < 0.05 [all *p* values corrected for multiples comparisons (Bonferroni)]. Numbers on the *X*-axis refer to blocks of the “circuit game.”

Furthermore, in the nine stroke patients who received dual-tDCS as the first Intervention, statistical analysis disclosed a carry-over effect on the Baseline performance (PI) 1 week after the first Recall session, i.e., 2 weeks after dual-tDCS. Thus, their second Baseline PI (sham session) was better than the first Baseline PI (dual-tDCS session); this fits with a carry-over effect and further reinforces the idea of lasting generalization of performance improvement. Could this carry-over effect have induced a ceiling effect during the second (sham) intervention or have skewed the main outcome measure, i.e., the comparison of the LI from Recall 1 between dual-tDCS and sham? Our contention is that the answer in negative for the following reasons. First, the statistical analyses on the primary outcome measure (the LI) did not demonstrate an order effect. Second, as can be appreciated from Figure A2 in Appendix and Appendix 2, during the second Intervention, the mean LI improved up to 9% 30 min after sham, which demonstrates that motor skill learning did not reach a ceiling. When comparing the two panels of Figure A2 in Appendix, it appears clearly that dual-tDCS improved online motor skill learning and long-term retention in both groups, and that motor skill learning also took place during and after sham, although to a much lesser extent. Third, even if a lasting carry-over effect and/or generalization were induced by dual-tDCS during the first Intervention, the stroke patients learned an *alternative* version of the circuit during the second Intervention (sham). It would thus be extremely surprising to observe a ceiling effect on this *new* motor skill, i.e., a *new* circuit of identical length and complexity but arranged in a different order. Anyway, if dual-tDCS indeed induced a ceiling effect, this would be an incredible achievement for neurorehabilitation: a single-session of dual-tDCS applied during motor skill learning would have brought these chronic stroke patients to the maximum of their motor potential! This seems very unlikely.

### Possible mechanisms underlying improvements induced by dual-tDCS

Several mechanisms may explain the dual-tDCS-induced improvement in motor skill learning and retention in stroke patients. First, dual-tDCS may have re-balanced deregulated interhemispheric interactions. According to the hypothesis of interhemispheric rivalry, deregulated interhemispheric interactions influence residual paretic hand function in stroke patients (Murase et al., 2004). Both rTMS and tDCS have the potential to re-balance these abnormal interhemispheric interactions and to improve motor performances (Hummel et al., 2005; Nowak et al., 2009). In healthy volunteers, dual-tDCS increases excitability on the anodal side associated with a decrease of excitability on the cathodal side (Mordillo-Mateos et al., 2012). A recent study has demonstrated that dual-tDCS could also re-balance abnormal interhemispheric interaction by inducing both a reduction of cortical excitability in the contralesional hemisphere, and an augmentation of excitability in the ipsilesional hemisphere associated with a significant reduction of the transcallosal inhibition from the contra to the ipsilesional hemisphere (Bolognini et al., 2011). Moreover, anodal tDCS applied over the ipsilesional M1 and cathodal tDCS applied over the contralesional M1 both improved fMRI activation during paretic hand movements proportionally to motor function improvement, including enhanced activation of the ipsilesional M1 and connected premotor areas (Stagg et al., 2012). Beyond inducing changes in neuronal membrane excitability, tDCS can modulate glutamatergic and γ-aminobutyric acid (GABA) systems in the motor cortex (Nitsche and Paulus, 2000; Nitsche et al., 2005; Stagg et al., 2009). These modulations are particularly relevant for motor skill learning and post-stroke neurorehabilitation, as therapeutic manipulation of the glutamatergic and GABAergic systems in the perilesional motor cortex enhances functional recovery in mice after stroke (Clarkson et al., 2010, 2011). The beneficial effects driven by modulation of the glutamatergic system may ultimately lead to the release of BDNF (Clarkson et al., 2011). It is worth noting that tDCS increases BDNF secretion and synaptic plasticity in animals (Fritsch et al., 2010), which could be a key mechanism underlying tDCS-induced improvements (Reis et al., 2009; Krakauer et al., 2012). Such tDCS-induced modulations of cortical excitability and molecular environment may also underlie the generalization observed in the current study.

Second, whereas M1 was targeted bilaterally, the current flow delivered by tDCS is not very focal and likely spread to the adjacent dorsal premotor cortex (PMd) and S1. From a neurophysiological point of view, exquisitely focal stimulation is undoubtedly superior. However, from a neurorehabilitation point of view, concomitant stimulation of adjacent cortical areas by tDCS may well be beneficial, since both PMd and S1 are involved in motor skill learning and post-stroke recovery and can be modulated by non-invasive brain stimulation to enhance motor skill learning (Meehan et al., 2011a; Kantak et al., 2012). Furthermore, the effects of tDCS are not circumscribed to the cortical area under the electrodes, but also involve distant interconnected areas (Stagg et al., 2012).

Third, there may be additional mechanisms specific to dual-tDCS due to (i) a synergic effect of dual stimulation over M1 bilaterally, (ii) a different current flow direction compared to classical tDCS approach, and (iii) additional effects on interconnected areas. The hypothetical existence of mechanisms specific to dual-tDCS remains to be tested.

Finally, we speculate that dual-tDCS may have non-specifically modulated attention, fatigue, or motivation in stroke patients, though this was not formally tested. Despite allowing rest between blocks of Training, eight stroke patients showed online worsening under sham, suggesting a fatigue effect. Since none of the stroke patients worsened under dual-tDCS, dual-tDCS could have blocked global fatigue or muscle fatigue. Such an anti-fatigue effect has been suggested after tDCS (Cogiamanian et al., 2007) or cTBS (Ackerley et al., 2010). Alternatively, progressive worsening or lack of online improvement may reflect a progressive drift in attention, concentration and/or motivation. Since recent experiments re-emphasized the importance of reward and motivation in motor skill learning (Abe et al., 2011), a high score was displayed during the rest periods to lessen motivation or attention drifts. Moreover, although tDCS can maintain attention and motivation (Kang et al., 2009), electrodes in the current experiment were not placed over the prefrontal cortex, which mediates these functions.

### Limitations

This experiment has some limitations. First, most of the stroke patients presented mild to moderate disability (mRS 1–3), although some of them had poor residual digital dexterity and/or bimanual ability (see Baseline PPT and ABILHAND scores, Table 1). Nevertheless, all showed training-induced improvement. Thus, whether dual-tDCS improves motor skill learning in severely impaired stroke patients remains to be tested. In fact, dual-tDCS or cathodal tDCS of the contralesional M1 might be deleterious in the most severely impaired stroke patients. Indeed, worsening of paretic upper limb performance has been observed in severely impaired stroke patients after inhibitory stimulation of the contralesional hemisphere with cathodal tDCS (Bradnam et al., 2012) or cTBS (Lotze et al., 2006; Hummel et al., 2008; Ackerley et al., 2010). However, in the current study, dual-tDCS did not cause worsening on any tasks performed with the paretic upper limb, in line with previous reports (Lindenberg et al., 2010; Zimerman et al., 2012). Moreover, dual-tDCS did not worsen the non-paretic hand function. To confirm the potential therapeutic impact of dual-tDCS, a multicentre randomized control trial with a larger range of impairments is required. Furthermore, even if the “circuit game” involved the whole upper limb, no clinical scales like the Fugl-Meyer or Wolf Motor Function tests were used, and these should also be tested in a large trial.

A second limitation was that the stroke patients were relatively heterogeneous in terms of stroke localization (cortical, subcortical, brainstem), mechanisms (large cortical or subcortical strokes, lacunar infarcts, hemorrhage), and presence of additional vascular injuries. However, this relative heterogeneity may also be a strength, as this cohort matches “real-life” stroke patients, emphasizing the potentially wide therapeutic impact of dual-tDCS.

Third, this study was based on the premise that dual-tDCS could re-balance abnormal interhemispheric interactions that are known to impair post-stroke recovery (Murase et al., 2004; Bolognini et al., 2011; Stagg et al., 2012). However, no measures of cortical excitability with TMS or changes in activation pattern with fMRI were performed.

Finally, potential confounding effects due to attention, fatigue, concentration, and blinding were not evaluated. However, we feel it unlikely that such non-specific effects might by themselves explain the intensity and nature of the improvements observed in motor skill learning, given the localization of the electrodes and the demonstration that blinding with sham tDCS is efficient (Gandiga et al., 2006).

## Conclusion

This is the first demonstration that a single-session of dual-tDCS applied during training dramatically enhanced online motor skill learning with the paretic hand in stroke patients, which translated into successful long-term retention of the motor skill. Remarkably, dual-tDCS enhanced the *quality* of motor skill learning by increasing the shift of the speed/accuracy trade-off. Furthermore, the combination of motor skill learning and dual-tDCS led to a generalization of motor performance improvements in the paretic hand, without concomitant worsening in the non-paretic hand. Finally, recalling the motor skill learned under dual-tDCS after 1 week may place the motor system in an optimal state for subsequent improvements in motor skill learning or in complex tasks. This generalization is particularly attractive in the context of neurorehabilitation. Further studies with TMS and fMRI should explore the mechanisms underlying these improvements mediated by dual-tDCS, as well as whether repeated training sessions combined with dual-tDCS lead to cumulative improvement.

## Conflict of Interest Statement

The authors declare that the research was conducted in the absence of any commercial or financial relationships that could be construed as a potential conflict of interest.

## Appendix 1

Since the motor skill has been designed to involve a speed/accuracy trade-off, both velocity and error need to be combined into a single parameter that reflects performance improvement (or worsening) over time, the Learning index (LI; Lefebvre et al., 2012). The computation of the LI requires combining velocity and error values for each subject at each block. By definition, the LI has been designed to increase with improvements in both speed and error, or when one parameter improves and the other does not deteriorate.

The circuit game was displayed and analyzed with a dedicated software which expresses error and velocity with arbitrary grid unit (*u*) as *u*/s for velocity and *u*^2^ for error. One arbitrary grid unit (u) displayed on the computer screen is equivalent to a distance of 0.3 cm traveled through in straight line by the computer mouse. Typically, error values ranges from 0.01 to 1.2 *u*^2^ and velocity values ranges from 1 to 14 *u*/s. Thus, in order to compute an index (i) combining these two parameters expressed with different units and (ii) not skewed by the much greater size of velocity compared to error, the error and velocity were normalized as Pe and Pv. Thus, Pe and Pv were obtained for each block of each stroke patient with the following formula:

$$\begin{array}{rcll} Pe & =\text{a}∕patienterror; & & \\ Pv & =patientspeed∕\text{b.} & & \end{array}$$

This normalization has been performed using a constant term for velocity (*b*) and for error (*a*) obtained on a group of seven other stroke patients while they trained to perform the “circuit game” in a pilot experiment.

$$\begin{array}{rcll} a & =0.371\;u^{2} & & \\ b & =4.858\;u∕\text{s} & & \end{array}$$

Next, Pe and Pv were combined to calculate the PI for each block of each patient:

PI=Pe×Pv

Finally, the evolution of performance as a percentage from Baseline (i.e., the LI) was calculated with the formula:

LI=[(PI−PI baseline)/PI baseline]×100

Since the LI (i.e., the main outcome measure) is calculated with the formula LI = [(PI − PI baseline)/PI baseline] × 100, individual normalization of performance improvement over time was embedded in the calculation.

Since “*a*” and “*b*” are constant values, the normalization of error and velocity could have been calculated with any arbitrary value. Nevertheless, we decided to derive “*a*” and “*b*” constants from real data obtained in seven pilot stroke patients, having thus a straightforward behavioral significance.

Finally, as depicted in Figure 2, the familiarization was performed on a very simple circuit (a square) with a much lower level of complexity, which cannot be compared with the more complex circuits used for motor skill learning. The goal of the familiarization was to allow the stroke patients to be acquainted with the task, to try the computer mouse, to warm up a little bit i.e., to be familiarized with the setup and the task. Therefore, we did not put emphasis on error and speed during familiarization.

## Appendix 2

### Additional analyses of the data from the first session only (“parallel-group design”)

We performed additional analyses with the data from the first session (Intervention and Recall) only, i.e., as if the study had assumed a parallel-group design, with nine stroke patients randomly allocated to real-dual-tDCS and the nine others to sham (see Figure A2).

Baseline characteristics were identical for age (*p* = 0.9), lesion localization (cortical/subcortical, *p* = 0.62), mRS (*p* = 0.1), ABILHAND (*p* = 0.6), PPT (*p* = 0.37), MaxHF (*p* = 0.73), and Baseline PI (*p* = 0.22).

At Recall 1, the LI was statistically superior 1 week after real-dual-tDCS (52% ± 25) than after sham (7% ± 30; *p* = 0.006), as well as for Recall 2 (*p* = 0.006), demonstrating that the long-term retention of the learned motor skill was superior after real-dual-tDCS than after sham.

The rmANOVA on the LI during Training and up to 60 min after showed a significant “Time” × “Stimulation” interaction (*p* = 0.017) suggesting that dual-tDCS led to greater online motor skill learning and superior Early Recalls than sham. rmANOVA also showed a significant effect of “Stimulation” (*p* = 0.026), suggesting that dual-tDCS-induced a greater online motor skill learning since the fifth block of Training (*p* = 0.046) and a significant effect of “Time” (*p* < 0.001). *Post hoc* analyses demonstrated that dual-tDCS led to a significantly greater and more rapid improvement than sham.

Both cross-over and parallel-group design have advantages and drawbacks. We believe that using the stroke patients as their own controls in this cross-over study (as done in previous studies; Kim et al., 2006; Zimerman et al., 2012) is not problematic since the stroke patients trained on *different* circuits (of identical length and complexity), i.e., learned a *different* skill at each session. Since statistical analyses did not demonstrate an order effect (see Result), the cross-over design allowed us to further characterize the positive effect of dual-tDCS on online motor skill learning and long-term retention, especially by demonstrating an improved shift of the speed/accuracy trade-off after dual-tDCS in the same stroke patients.

**Table A1:** **Individual changes in motor skill learning, expressed as percentage change from Baseline, for velocity, error, and learning index (LI) for sham (A) and dual-tDCS (B). Numbers 1–18 correspond to the 18 stroke patients in the same order as in Table 1**.

Behavioral results of motor skill learning															
	End of “training” period			“After” period			“After 60 min” period			“Recall 1”			“Recall 2”		
	Velocity	Error	LI	Velocity	Error	LI	Velocity	Error	LI	Velocity	Error	LI	Velocity	Error	LI
	(%)	(%)	(%)	(%)	(%)	(%)	(%)	(%)	(%)	(%)	(%)	(%)	(%)	(%)	(%)
**A**															
1	−23	−39	21	−10	−34	35	−11	−30	27	−26	−33	4	−13	−36	29
2	−16	−24	19	−23	34	−36	−26	−9	−1	−8	−5	5	−12	2	−1
3	13	7	3	−22	−24	4	−5	−11	7	5	−6	11	13	−4	17
4	−32	−25	13	−41	−42	27	13	−17	44	11	−18	34	−14	−28	28
5	−43	−34	6	−36	−29	1	−29	−26	13	−22	−26	18	−23	−24	11
6	4	16	−7	−10	−7	−1	−34	4	−32	−36	−19	−17	−38	−6	−30
7	17	96	−14	6	135	−50	22	122	−35	36	147	−45	91	176	−23
8	−21	−13	−8	−20	−15	−1	−25	−22	5	−29	−20	−6	−25	10	−34
9	−44	−29	−17	−51	−36	−6	−15	−12	−7	−48	−31	−19	−54	−38	−16
10	−4	−26	25	19	−23	44	31	−26	60	2	−26	47	1	−36	52
11	19	−6	15	15	−3	10	16	−10	18	1	−10	5	8	−11	14
12	−8	−8	−4	−1	−18	17	4	−9	9	36	6	15	34	9	30
13	62	25	41	78	21	54	104	15	78	74	51	18	76	25	48
14	100	−14	32	15	−44	61	40	−44	53	89	−2	42	63	−35	54
15	−30	5	−27	−33	1	−30	−27	8	−27	−15	11	−12	−27	4	−23
16	−15	15	−22	−2	10	−7	−11	0	−4	22	30	−3	3	18	−10
17	18	−3	23	14	1	16	28	6	19	27	12	10	26	3	20
18	−26	−7	−21	−36	−1	−34	−39	−12	−31	−54	−26	−30	−52	−20	−43
Mean	−2	−4	4	−8	−4	6	2	−4	11	4	2	4	3	1	7
SD	36	31	20	30	41	31	35	35	33	39	42	24	42	48	31
**B**															
1	12	−40	102	10	−34	137	22	−27	77	33	−20	77	37	−33	119
2	40	−4	49	23	3	−26	32	−15	55	8	10	5	24	−3	30
3	103	7	94	97	13	37	97	18	67	101	26	62	104	22	68
4	−25	−47	65	−28	−39	20	−31	−33	25	9	−42	96	−2	−37	68
5	13	−14	17	44	−20	64	17	−8	9	39	−23	47	39	−26	58
6	39	−1	46	34	9	19	121	15	98	63	−1	67	112	−2	138
7	23	9	10	−11	3	−1	83	9	54	86	60	2	46	42	2
8	−14	−24	31	−17	−27	9	−23	−33	31	−2	−18	44	−7	−26	45
9	42	−3	51	54	2	26	63	3	76	49	−3	50	40	13	43
10	24	−33	81	25	−27	31	29	−16	55	36	−15	62	38	−21	70
11	90	5	78	103	−12	121	68	9	63	73	13	48	210	57	101
12	18	−1	26	22	3	8	14	14	1	8	−2	12	6	−7	22
13	18	−1	17	18	−2	15	16	−6	24	15	−13	37	24	−17	56
14	0	−29	47	−1	−28	−10	27	−19	60	−2	−30	39	22	−27	75
15	33	11	30	33	18	−2	35	12	27	63	10	63	59	6	54
16	−18	−31	15	−10	−28	37	−17	−36	31	7	−20	32	−6	−17	16
17	32	−1	14	20	−2	42	29	−14	31	24	−16	34	24	−16	34
18	−24	−31	19	−27	−23	13	−29	−30	13	4	−9	14	1	−16	26
Mean	23	−13	44	22	−11	30	31	−9	44	34	−5	44	43	−6	57
SD	35	19	29	37	18	42	43	19	27	32	23	25	53	26	36

*A positive percentage change reflects increased velocity, decreased error, or improved LI. End of the training period corresponds to the last five blocks of the training period*.

## Author Note

Trial registration: The trial protocol has been registered on http://clinicaltrials.gov/ under the number NCT01519843.
